# Supplementary material for: Vitamin D Modified DSS-Induced Colitis in Mice via STING Signaling Pathway
Source: Biology (Basel). 2025 Jun 18;14(6):715. doi: 10.3390/biology14060715 (PMC12190092; doi:10.3390/biology14060715)
Supplement: Supplementary file 1 [file biology-14-00715-s001.zip › Supplementary Materials S1: 16s-report of gut microbiota/Groups/diff_analysis/TukeyHSD/Tukey_Genus_rotate.pdf]

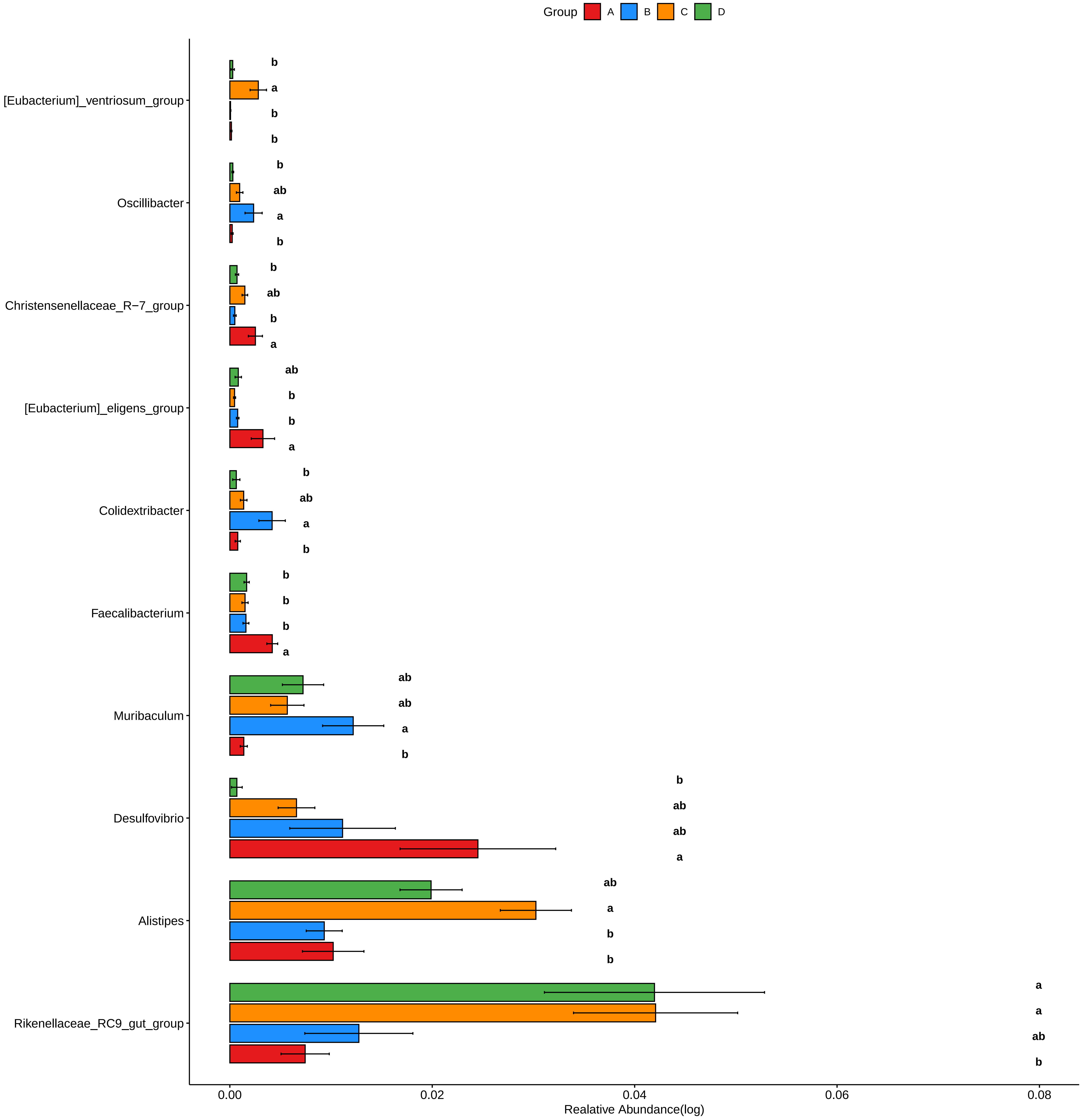
Group
A
B
C
D
[Eubacterium]_ventriosum_group
Oscillibacter
Christensenellaceae_R−7_group
[Eubacterium]_eligens_group
Colidextribacter
Faecalibacterium
Muribaculum
Desulfovibrio
Alistipes
Rikenellaceae_RC9_gut_group
0.00
0.02
0.04
0.06
0.08
Realative Abundance(log)
